# Avian Influenza A(H10N7) Virus–Associated Mass Deaths among Harbor Seals

**DOI:** 10.3201/eid2104.141675

**Published:** 2015-04

**Authors:** Rogier Bodewes, Theo M. Bestebroer, Erhard van der Vries, Josanne H. Verhagen, Sander Herfst, Marion P. Koopmans, Ron A.M. Fouchier, Vanessa M. Pfankuche, Peter Wohlsein, Ursula Siebert, Wolfgang Baumgärtner, Albert D.M.E. Osterhaus

**Affiliations:** Erasmus Medical Centre, Rotterdam, the Netherlands (R. Bodewes, T.M. Bestebroer, E. van der Vries, J.H. Verhagen, S. Herfst, M.P. Koopmans, R.A.M. Fouchier, A.D.M.E. Osterhaus);; University of Veterinary Medicine, Hannover, Germany (V.M. Pfankuche, P. Wohlsein, U. Siebert, W. Baumgärtner, A.D.M.E. Osterhaus);; Artemis One Health, Utrecht, the Netherlands (A.D.M.E. Osterhaus)

**Keywords:** influenza A virus, harbor seals, H10N7, viruses, Germany, influenza, Phoca vitulina

**To the Editor:** Avian influenza A viruses occasionally cross the species barrier; influenza A(H5N1) virus and the recently emerged influenza A(H7N9) virus are prime examples of bird-to-human transmission (*1*,*2*). In addition, avian influenza A viruses can cross to various other mammalian species, including pinnipeds (e.g., seals) (*3*,*4*).

Recently, mass deaths have occurred among harbor seals (*Phoca vitulina*); hundreds of carcasses washed up the shores of Sweden (March 2014), Denmark (July 2014), and Germany (October 2014). Approximately 1,400 dead harbor seals were seen in the coastal waters of Schleswig-Holstein in Germany alone, where the population is ≈12,000 animals.

We report the investigation of the deaths of 17 seals from different age groups that were found dead on the islands of Helgoland and Sylt, Germany, during the second week of October 2014. Complete necropsies were performed on the carcasses, which were in variable nutritional conditions, ranging from very poor to good. Necropsy results showed consistently poorly retracted lungs with severe congestion, occasional diffuse consolidation, and multifocal firm nodular areas of gray-yellow discoloration with varying numbers of metazoic parasites. Histologic examinations showed acute necrotizing bronchitis and adenitis of bronchial glands with sloughing of epithelial cells (Figure, panel A). Occasionally, mild interstitial pneumonia was found. Multifocal pyogranulomatous to necrotizing pneumonia was associated with parasite infestation. A few animals had suppurative to necrotizing or nonsuppurative rhinitis and tracheitis.

**Figure:**
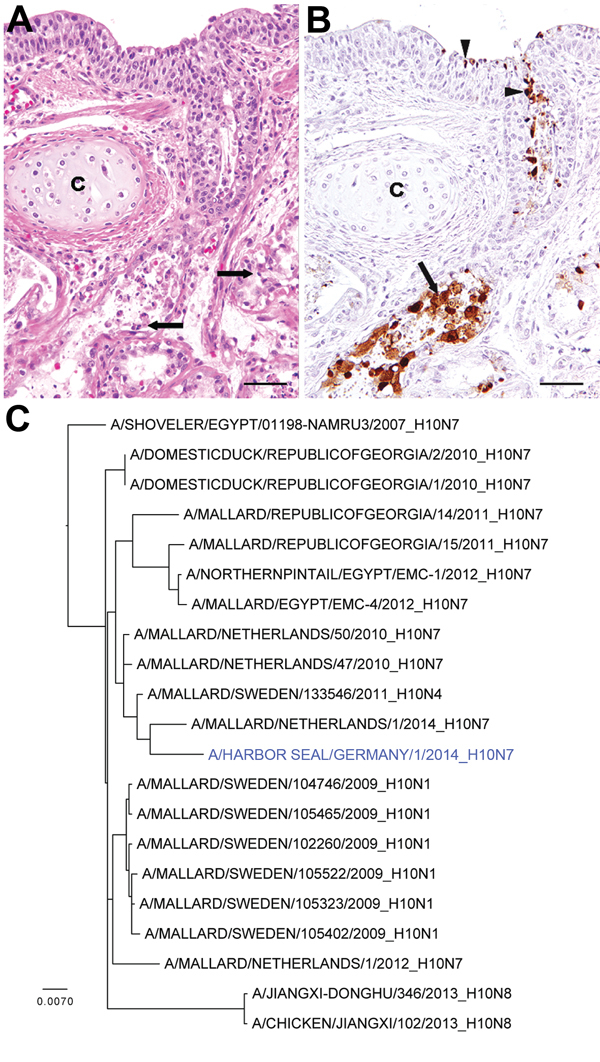
Histopathologic and phylogenetic analyses of necropsy samples from harbor seals infected with avian influenza A(H10N7) virus, Germany, 2014. A) Lung of harbor seal showing marked necrosis and sloughing of epithelial cells in bronchial glands (arrows); c = bronchial cartilage; hematoxylin and eosin stain. Scale bar indicates 50 μm B) Immunohistochemical labeling of influenza A nucleoprotein in bronchial epithelial cells (arrowheads) and glandular epithelial cells (arrows); c = bronchial cartilage; avidin-biotin-peroxidase complex method. Scale bar indicates 50 μm. C) Maximum-likelihood phylogenetic tree of the partial hemagglutinin gene (1,577 nt) of the influenza A/harbor seal/Germany/1/2014 (H10N7) isolate and various other closely related viruses. GenBank accession numbers are provided in Technical Appendix Table 1. Scale bar indicates nucleotide substitutions per site.

Because mass deaths among seals were caused by phocine distemper virus in the same area in 1988 and 2002, we tested lung and throat swab samples for morbillivirus using reverse transcription PCR (RT-PCR) and immunohistochemical analysis (*5*). In addition, real-time RT-PCR targeting the influenza A virus matrix gene was performed (*6*). No indications for the morbillivirus were detected by RT-PCR and immunohistochemistry; however, in lung lesions and throat swab samples of 11 animals, a positive signal was observed by the influenza A matrix gene real-time RT-PCR (cycle threshold values 15.0–33.9). Influenza A virus (A/harbor seal/Germany/1/2014) was subsequently isolated from lung and throat swab samples; the virus replicated to high titers in 11-day-old embryonated chicken eggs and on MDCK cells. By PCR using specific primers and subsequent Sanger sequencing of the hemagglutinin and neuraminidase genes, this virus was characterized as an influenza A virus of the H10N7 subtype, commonly found in migratory waterfowl (*6*). In addition, genetic analyses of all other gene segments indicated that the influenza virus A/harbor seal/Germany/1/2014 was most closely related to various influenza A viruses detected in wild birds. Specifically, the hemagglutinin and neuraminidase genes were genetically most closely related to subtype H10N7 viruses recently found in migratory ducks in Georgia, Egypt, and the Netherlands (Figure, panel C) (*7*). Genetic analyses were based on BLAST analyses using public databases available as of October 17, 2014 (http://www.ncbi.nlm.nih.gov, http://www.gisaid.com) and supplemented with H10 and N7 sequences from the international wild bird surveillance program of Erasmus Medical Center (Technical Appendix Table). A maximum-likelihood phylogenetic tree of the hemagglutinin gene was generated by using PhyML version 3.1 (*8*) with the general time reversible +I+Γ model of nucleotide substitution; a full heuristic search and subtree pruning and regrafting searches were performed. The tree was visualized by using Figtree version 1.4.0 (http://tree.bio.ed.ac.uk/software/figtree).

To further elucidate the role of influenza A(H10N7) virus in the pathogenesis of the disease causing deaths among the seals, we conducted immunohistochemical analysis on the lungs using an influenza A virus nucleoprotein-specific monoclonal antibody (*9*). Evaluation of the lung tissues of the dead seals showed viral antigen in cytoplasm and nuclei of epithelial cells of bronchi and bronchial glands of affected lung areas (Figure, panel B), which suggests that this virus played a major role in the deaths. Immunohistochemical analysis performed on various organs (including brain and olfactory bulb) indicated that viral antigen was restricted to the respiratory tract.

Although avian influenza A virus infections previously have caused mass deaths in seals (*3*,*4*,*10*), subtype H10N7 has not been associated with such events. We can speculate that the ongoing deaths could eventually affect all harbor seal populations of northwestern Europe and have consequences for wildlife management and seal rehabilitation activities. In addition, preliminary analysis of the hemagglutinin sequence of the influenza A(H10N7) virus suggests the presence of molecular determinants that indicate mammalian adaptation. Various analyses are ongoing to answer questions about the route of transmission among seals and possible transmissibility to humans.

Note added in proof: Zohari et al. also recently reported the involvement of avian influenza A(H10N7) virus in mass deaths of harbor seals in Sweden (Euro Surveill. 2014;19:pii: 20967).

Technical Appendix Details of hemagglutinin sequences shown in the Figure.
