# Supplementary material for: Avian Influenza A(H10N7) Virus–Associated Mass Deaths among Harbor Seals
Source: Emerg Infect Dis. 2015 Apr;21(4):720–2. doi: 10.3201/eid2104.141675 (PMC4378483; doi:10.3201/eid2104.141675)
Supplement: Technical Appendix — Details of hemagglutinin sequences shown in the Figure. [file 14-1675-Techapp-s1.pdf]

Article DOI: http://dx.doi.org/10.3201/eid2104.141675

Avian Influenza A(H10N7) Virus–Associated Mass Deaths among Harbor Seals

Technical Appendix

Technical Appendix Table. Details of hemagglutinin sequences shown in the Figure*

Isolate name	Accession no.	Online database
A/seal/Germany/EMC-1/2014(H10N7)	EPI544351	GISAID EpiFlu
A/mallard/Netherlands/1/2014(H10N7)	EPI552751	GISAID EpiFlu
A/mallard/Sweden/133546/2011(H10N4)	CY183991.1	GenBank
A/domestic duck/RepublicofGeorgia/2/2010(H10N7)	CY185457.1	GenBank
A/domestic duck/RepublicofGeorgia/1/2010(H10(N7)	CY185449.1	GenBank
A/mallard/Sweden/104746/2009(H10N1)	CY183855.1	GenBank
A/mallard/Sweden/105465/2009(H10N1)	CY183927.1	GenBank
A/mallard/Sweden/102260/2009(H10N1)	CY183847.1	GenBank
A/mallard/Sweden/105323/2009(H10N1)	CY183887.1	GenBank
A/mallard/Sweden/105402/2009(H10N1)	CY183911.1	GenBank
A/mallard/Sweden/105522/2009(H10N1)	JX566079.1	GenBank
A/mallard/Netherlands/50/2010(H10N7)	EPI552752	GISAID EpiFlu
A/mallard/Netherlands/47/2010(H10N7)	EPI552753	GISAID EpiFlu
A/mallard/Republic of Georgia/14/2011(H10N7)	CY185689.1	GenBank
A/mallard/Republic of Georgia/15/2011(H10N7)	CY185385.1	GenBank
A/northern pintail/Egypt/EMC-1/2012(H10N7)	EPI552754	GISAID EpiFlu
A/mallard/Egypt/EMC-4/2012(H10N7)	EPI552755	GISAID EpiFlu
A/mallard/Netherlands/1/2012(H10N7)	EPI552756	GISAID EpiFlu
A/shoveler/Egypt/01198-NAMRU3/2007(H10N7)	EPI372402†	GISAID EpiFlu
A/Jiangxi-Donghu/346/2013(H10N8)	EPI497477‡	GISAID EpiFlu
A/chicken/Jiangxi/102/2013(H10N8)	EPI530542§	GISAID EpiFlu

*We gratefully acknowledge the authors, originating and submitting laboratories of the sequences from the Global Initiative on Sharing Avian Influenza Data (GISAID) EpiFluTM database on which this research is based. All submitters of data may be contacted directly through the GISAID website.
†Originating laboratory: US Naval Medical Research Unit No.3, Egypt; Submitting laboratory: Centers for Disease Control and Prevention, Atlanta, GA, USA (*1*).
‡Originating and submitting laboratory: World Health Organization Chinese National Influenza Center, Beijing, China (*2*).
§Originating laboratory: South China Agricultural University, Guangzhou, China; Submitting laboratory: South China Agricultural University, Guangzhou, China (*3*).

References

1. Gerloff NA, Jones J, Simpson N, Balish A, Elbadry MA, Baghat V, et al. A high diversity of Eurasian lineage low pathogenicity avian influenza A viruses circulate among wild birds sampled in Egypt. PLoS ONE. 2013;8:e68522. PubMed http://dx.doi.org/10.1371/journal.pone.0068522

2. Chen H, Yuan H, Gao R, Zhang J, Wang D, Xiong Y, et al. Clinical and epidemiological characteristics of a fatal case of avian influenza A H10N8 virus infection: a descriptive study. Lancet. 2014;383:714–21. PubMed http://dx.doi.org/10.1016/S0140-6736(14)60111-2

3. Qi W, Zhou X, Shi W, Huang L, Xia W, Liu D, et al. Genesis of the novel human-infecting influenza A(H10N8) virus and potential genetic diversity of the virus in poultry, China. Euro Surveill. 2014;19:pii: 20841. PubMed
